# Wet Spinning of Sustainable Hydroxypropyl Cellulose Fibers

**DOI:** 10.1021/acs.biomac.5c02062

**Published:** 2025-11-25

**Authors:** Manon Guivier, Christoph Weder

**Affiliations:** a Adolphe Merkle Institute, Polymer Chemistry and Materials, University of Fribourg, Fribourg 1700, Switzerland; b NCCR Bio-inspired Materials, University of Fribourg, Fribourg 1700, Switzerland

## Abstract

Hydroxypropyl cellulose (HPC) is a biobased, biodegradable, and water-soluble material that is used as an emulsifier, thickener, and stabilizer in aqueous formulations. Its solid-state properties render HPC also attractive for water-soluble sanitary products, packaging, and biomedical applications. While HPC films and coatings are well-known, HPC fibers have hardly been investigated, arguably, due to the lack of methods to spin HPC fibers. Here, we show that fibers can readily be produced by wet spinning aqueous HPC solutions into an aqueous CaCl_2_ coagulation bath. This process induces significant alignment of HPC chains under shear forces, resulting in fibers with considerably higher stiffness (Young’s modulus = 1.3 GPa) and tensile strength (36 MPa) than solution-cast HPC films (Young’s modulus = 0.7 GPa, tensile strength = 19 MPa). The HPC fibers retain their mechanical integrity upon conditioning at 60% relative humidity, although stiffness is considerably reduced. Wet spinning is readily scalable and affords stiff and strong fibers that represent a promising alternative to woven and nonwoven synthetic fibers.

## Graphical abstract

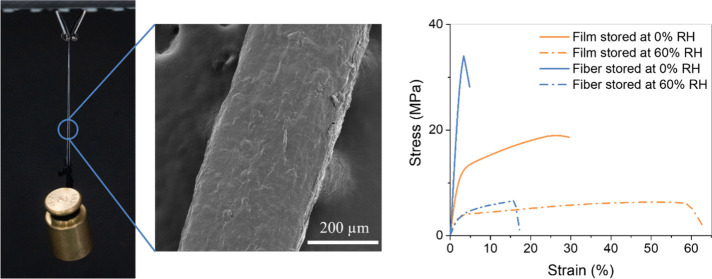

## 1 Introduction

With the objectives of increasing the sustainability of plastics and decreasing their dependence on petroleum-based feedstock,^1^ considerable research efforts are currently focused on the development of biobased and biodegradable polymers, offering reduced environmental footprints and controlled end of life.^2–4^ While the fraction of these materials in the market remains low, the potential to replace petroleum-based plastics in various industries, including packaging, sanitary products, and biomedical applications, is tremendous.^5–7^

Among other renewable polymers, cellulose and its derivatives have attracted significant interest due to their abundance and low cost, their biodegradability, and the widely tunable properties that can be achieved by chemical modification.^8^ Hydroxypropyl cellulose (HPC), a commercially available cellulose ether, is one example of a widely used cellulose derivative that is water-soluble and nontoxic. While HPC is mostly used as an emulsifier, thickener, and stabilizer in the food industry, personal care products, paints, and pharmaceutical products,^9–12^ the polymer also exhibits good film-forming ability and offers high mechanical flexibility, high optical transparency, and a relatively low oxygen permeability.^11,13–15^ These properties render HPC also suitable for applications in solid forms, e.g., dissolvable coatings and controlled-release agent in tablets and capsules.^16–18^

Recent studies have also explored HPC films and coatings as environmentally friendly alternatives to petroleum-based food packaging.^14,19^ Its high solubility in water also renders HPC potentially useful as a basis to produce dissolvable fibers, for example, for cosmetic products, wound dressing, or flushable products.^20–22^ The fibers can be used in both woven and nonwoven forms, serve as active and functional layers in multilayer architectures, or serve as reinforcing fillers in composites.^23–25^

Interestingly, however, reports on the processing of HPC into fibers are scarce. Shukla et al. reported electrospun fibers of HPC that were collected as randomly oriented mats and used as templates for the synthesis of tin oxide nanofibers.^26^ Qin and Xu reported the extrusion of methacrylated hydroxypropyl cellulose into cross-linked fibers that no longer display the water solubility of HPC.^27^ To the best of our knowledge, a simple, scalable process for HPC spinning into fibers that could be used as the basis for woven products has yet to be reported.

Here, we show that HPC fibers can readily be produced by wet spinning from aqueous solutions if a salt solution is employed as a coagulation bath. This approach enables the production of individual, high-quality HPC fibers and is readily scalable. We investigated the impact of various processing parameters on the thermal stability, moisture content, and mechanical properties of the fibers and compared their characteristics with those of solvent-cast HPC films. Our findings contribute to a deeper understanding of HPC processability and offer key elements to develop high-performance, sustainable fibers whose properties satisfy industrial needs.

## 2 Experimental Section

### 2.1 Materials

Hydroxypropyl cellulose (HPC) with a weight-average molecular weight (Mw) of 370,000 g·mol^–1^ was purchased from Sigma-Aldrich and stored in a desiccator prior to use to avoid residual water sorption. Sodium chloride (NaCl), calcium chloride (CaCl_2_), hydrochloric acid (HCl), acetone, 2-butanol, 2-propanol, and dimethylacetamide (DMAc) were purchased from Sigma-Aldrich and used as received.

### 2.2 Methods

#### 2.2.1 HPC Spinning-Dope Preparation

To prepare aqueous HPC solutions with concentrations of 5–20 wt %, the dried HPC powder (0.5–2.0 g) was gradually poured into vials filled with deionized water (10 mL) and the mixtures were stirred at 500 rpm overnight until HPC had completely dissolved. The resulting HPC dopes were stored for a maximum of 2 weeks prior to wet spinning.

#### 2.2.2 Wet Spinning

Wet spinning experiments were performed using a 5 mL glass syringe equipped with a stainless-steel needle (19-gauge, inner diameter of 0.686 mm, from Hamilton). Using a NE-300 Just Infusion syringe pump (SyringePump), the HPC dope was spun at a flow rate of 0.5 mL/min into a coagulation bath (40 cm long), as illustrated in Figure 1. The residence time of the fibers in the coagulation was ca. 5 min, before they were attached to the rotating drum collector (diameter of 7 cm) and collected with a rotating speed of ca. 25 rpm (wind-up speed of 9.1 cm/s). After the collection, the HPC fibers were rinsed with deionized water to remove residual salts. To this end, the fibers were maintained on the drum collector and deionized water was gently poured along the collector. The fibers were subsequently placed in an oven at 50 °C for 48 h to dry and then stored in a desiccator filled with dried silica gel at ca. 0% relative humidity (RH) prior to further characterization.

**Figure 1:**
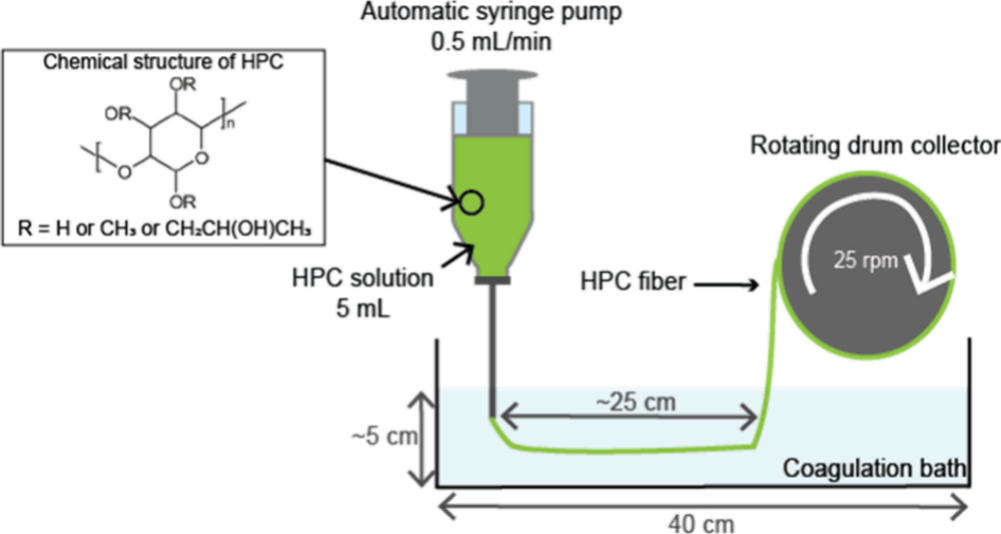
Schematic representation of the wet spinning process used to produce HPC fibers.

The processing parameters were optimized to produce continuous and homogeneous HPC fibers, and the various conditions that were explored are summarized in Table 1.

**Table 1:** Parameters Varied to Optimize the Wet Spinning of HPC Fibers

**parameters**	**range/type**
HPC concentration	5–20 wt %
coagulation bath	water, aqueous CaCl_2_ (2–8 wt %), aqueous NaCl (2–8 wt %), HCl (0.01–0.2M), 2-butanol, 2-propanol, acetone, DMAc:water (1:1 v/v)
temperature of the coagulation bath	25–80 °C

#### 2.2.3 Film Casting

Solvent-cast HPC films were prepared using aqueous solutions that were prepared as described above. The solutions with a concentration of 15 wt % (30 mL) were poured into poly­(tetrafluoroethylene) (PTFE) Petri dishes with a diameter of 9 cm. HPC films containing CaCl_2_ were prepared for mechanical tests, according to the same protocol. In this case, CaCl_2_ (0.4, 0.8, 1.6, or 2.4 g) was added to the HPC solutions (15 wt %, 100 mL), to produce HPC films with a target CaCl_2_ content in the dried films of 2.5, 5, 10, and 15 wt %, respectively, named HPC_CaCl2_X,_ X corresponding to CaCl_2_ content. The solutions were stirred for 4 h prior to casting into PTFE Petri dishes (approximately 30 mL of solution was poured into a PTFE Petri dish with a diameter of 9 cm). The solvent was allowed to evaporate under ambient conditions in a fume hood for 72 h. The films were then placed in an oven at 50 °C for an additional 48 h. In the case of films containing CaCl_2_, water accumulated on their surface, due to the presence of high salt concentration. This excess was gently removed with a tissue prior to storage in a desiccator filled with dried silica gel at ca. 0% RH. The average thickness of the films was measured with a digital micrometer (IP 65, Mitutoyo), measuring three films per composition and making seven measurements per film.

### 2.3 Characterization

#### 2.3.1 Moisture Uptake Measurements

Moisture uptake measurements of HPC fibers and films were performed using two desiccators containing sodium bromide and potassium bromide to reach relative humidities of 59.1 ± 0.4% and 81.7 ± 0.2% at 23 °C, respectively.^28^ Prior to measurements, all samples were stored in desiccators filled with dried silica gel at ca. 0% RH. For films, 1 × 1 cm^2^ squares were cut, while for fibers, bundles of six fibers (1 cm long) were prepared to ensure sufficient mass (ca. 404–420 mg dry sample weight) for accurate weighing. The initial dry weight (*M*_0_) was measured prior to storage at the designated relative humidities. The moisture uptake was then monitored gravimetrically by regularly weighing the sample until the equilibrium weight (*M*_eq_) was reached. The equilibrium moisture content was calculated according to eq 1, and reported values are the average of three measurements per type of sample.

$$\mathrm{Moisture}\;\mathrm{content}=\frac{M_{\mathrm{eq}}-M_{0}}{M_{0}}\times 100$$ 1

#### 2.3.2 Thermal Stability

Thermogravimetric analyses (TGA) of HPC fibers and films were performed using a Mettler Toledo TGA/DSC 1 STARe system. Analyses were conducted under a nitrogen atmosphere (50 mL/min) over a temperature range of 25–500 °C with a heating rate of 10 °C/min. Differential scanning calorimetry (DSC) analyses were realized using a Mettler Toledo DSC 5+ STAR system under a nitrogen atmosphere (60 mL/min). A heat–cool–heat program was performed with 8–10 mg samples in a 40 μL aluminum pan from −60 to 240 °C, with heating and cooling rates of 10 °C/min.

#### 2.3.3 Fiber Morphology and Diameter

Polarized optical microscopy (POM) was used to investigate the alignment of HPC chains in films and fibers. An Olympus BX51 microscope with an Olympus DPT2 camera was used in transmission mode. The samples were placed between two crossed linear polarizers (Olympus U-POT) to study their birefringence. Scanning electron microscopy (SEM) images of the surfaces and cross sections of fibers and energy-dispersive X-ray (EDX) spectra were acquired using a Tescan MIRA3 LM FE microscope. The following conditions were applied to record SEM images: an accelerating voltage of 5 kV and a working distance of ca. 20 mm. The fibers were first coated with a 4 nm-thick layer of Au (Cressington 208HR high-resolution sputter coater, U.K.). Images were analyzed with ImageJ software (v 1.53 t), and the average fiber diameter was determined from the images of nine individual fibers. EDX spectra were acquired using the following conditions: an accelerating voltage of 15 kV and a working distance of ca. 10 mm. The results were collected using AZtec software.

#### 2.3.4 Degree of Crystallinity

The degree of crystallinity of HPC fibers and films was determined from wide-angle X-ray scattering (WAXS) patterns. WAXS measurements were performed using a NanoMax-IQ camera (Rigaku Innovative Technologies) equipped with a Cu target sealed tube source (λ = 1.54 Å) (MicroMax-003 microfocus, Rigaku). Scattering data were recorded by a PILATUS 100K detector (Dectris), and the samples were maintained under vacuum during analysis. The sample-to-detector distance was calibrated using silver behenate. Before evaluation, the background was subtracted from the diffraction patterns. The degree of crystallinity (D.o.C.) was calculated using the integrated intensity under the crystalline peaks (*I*_c_) and the integrated intensity under the complete WAXS traces (i_trace_), according to eq 2.^29,30^

$$D.o.C.=\frac{I_{c}}{I_{trace}}\times 100$$ 2

#### 2.3.5 Mechanical Properties

The mechanical properties of HPC fibers and films were characterized by tensile tests conducted on a Zwick/Roell Z010 testing machine (10 kN) equipped with a 200 N load cell and 2.5 kN clamps. Fiber diameters were measured prior to any mechanical test, and each fiber was removed individually from a desiccator to minimize variations in moisture content. Two storage conditions were investigated: 0% RH and 60% RH. Desiccators filled with silica gel and sodium bromide, at 23 °C, were used to create dry and 60% RH environments, as mentioned above. For fibers, samples measuring 2.5 cm in length were tested. Films (thickness of ca. 200 μm) with fixed width and length of 5.4 mm and 2 cm, respectively, were cut with a die-cutting tool. All tests were performed at a strain rate of 10 mm/min and with a preload force of 0.005 N. The reported results are averages of five independent measurements, and errors are standard deviations.

Dynamic mechanical analysis (DMA) was performed for fibers and films stored at 0% RH using a TA Instruments Model Q800 DMA in tensile mode. Measurements were conducted over a temperature range of −80 to 150 °C, with a heating rate of 3 °C/min, a frequency of 1 Hz, and a strain amplitude of 0.1%. Fibers with a length of ca. 1 cm and films (thickness of ca. 200 μm) with a width of 5.4 mm and a length of 1 cm were tested. The reported results are averages of three independent measurements, and errors are standard deviations.

## 3 Results and Discussion

### 3.1 Optimization of the Wet Spinning Process

Wet spinning experiments were carried out using the setup shown in Figure 1. Different processing parameters were varied with the objective of optimizing the process to produce homogeneous and defect-free fibers that neither shrink nor dissolve in the coagulation bath. Based on preliminary screening experiments performed at room temperature, a spinneret with a fixed diameter of 0.686 nm and a flow rate of 0.5 mL/min were determined as suitable and these parameters were subsequently kept for all experiments. The spinneret diameter was selected to produce fibers with a diameter between 0.3 and 0.6 mm, while the flow rate was chosen to provide the extruded fibers with sufficient time to coagulate and to match the rotating drum collector speed. The rotating drum collector was used at its minimal speed, 25 rpm, corresponding to 9.1 cm/s, to avoid strong stretching of fibers during collection, which could lead to fibers with inhomogeneous diameters.

The parameters varied include the HPC concentration and the nature and temperature of the coagulation bath (Table 1). Table S1 (Supporting Information) details the various combinations of parameters that were explored and the corresponding outcomes.

We first focused on the concentration of the spinning dope, which affects the spinning process in a manner that is largely independent of the composition of the coagulation bath. At room temperature, HPC solutions with a concentration of 5 or 8 wt % had a viscosity that was too low to form homogeneous fibers, whereas solutions with a concentration of 15 or 20 wt % were too viscous, at least for the spinning equipment utilized here. A dope with an HPC concentration of 10 or 12 wt % was found to offer stable spinning conditions and produce homogeneous and defect-free fibers.

Among the various nonsolvents explored as coagulation baths, aqueous HCl solutions and alcohols (2-butanol and 2-propanol), at 23 °C, led to significant fiber shrinkage after collection on the rotating drum collector, despite the low take-up speed. The effect was likely caused by slow coagulation and drying, leading to an inhomogeneous moisture gradient and chain relaxation. Similar shrinkage and curling of wet-spun fibers have been observed in cellulose-based papers^31^ and TEMPO-oxidized cellulose nanofibers^32^ and were attributed to the relaxation of polymer chains in the coagulation bath. DMAc:water coagulation baths at 23 and 50 °C failed to coagulate HPC fibers even after extended immersion (2 h), making the collection of fibers impossible. In contrast, successful wet spinning of HPC fibers was achieved with coagulation baths based on heated aqueous salt solutions, especially when calcium chloride (CaCl_2_) was utilized. Indeed, literature reported the successful coagulation of cellulosic fibers into salt coagulation baths, as salts can influence the temperature-induced phase transition of thermosensitive polymers such as HPC.^33–36^ In our study, NaCl and CaCl_2_ baths successfully coagulated HPC fibers; however, the use of a NaCl bath led to inconsistent outcomes; some fibers could be collected while others could not be removed from the bath, reflecting poor reproducibility. Moreover, the salt concentration was found to affect the success of fiber coagulation. We found that a minimum of 4 wt % is required to obtain homogeneous fibers with a stable behavior once collected. Increasing the salt concentration slightly reduced the coagulation time required to obtain homogeneous fibers but could introduce a higher residual salt content in the fibers, necessitating multiple rinsing steps. Finally, the influence of the coagulation bath temperature was investigated, as HPC presents a lower critical solution temperature (LCST) of ca. 41 °C in deionized water. Therefore, the use of a coagulation bath above this temperature could induce aggregation and coagulation of HPC.^37,38^ Indeed, when a higher temperature was used (70 °C), the HPC fibers solidified nicely in water, but without salt, they reverted to a viscous liquid upon collection outside of the bath, where the temperature dropped back to RT. Thus, after exploring these parameters, a CaCl_2_ coagulation bath with a concentration of 4 wt % at a temperature of 70 °C and an HPC concentration of 12 wt % was identified as an optimal combination of conditions.

The collected HPC fibers were gently rinsed with deionized water to remove the residual salt from the fibers. The detailed conditions (see Section 2 for details) were empirically determined with the goal of removing the salt without dissolving the fibers. After applying this protocol, no residual salt was observed at the surface of HPC fibers, as reported in Figure 2.

**Figure 2:**
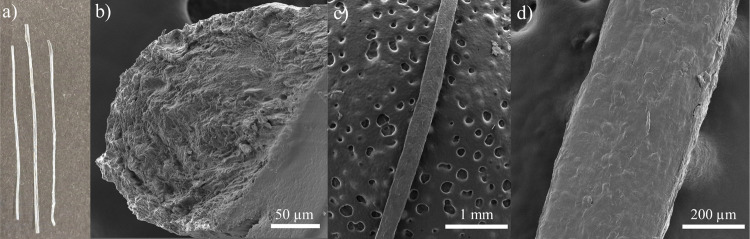
(a) Photograph of wet-spun HPC fibers. (b–d) Scanning electron microscopy images of the HPC fiber (b) cross-section and (c) surface, and (d) a magnified view of the fiber surface.

The morphology of the transparent fibers (Figure 2a) was characterized by scanning electron microscopy (SEM). The SEM images reveal the absence of pores in the fibers (Figure 2b) and a smooth surface without defects and observable salt residues (Figure 2c,d). Their analysis revealed an average fiber diameter of 512 ± 34 μm. The porous background observed in Figure 2c is due to the Au coating on the carbon tape used to stick the fiber on the metallic support and does not interfere with the sample structure. For comparison, we also prepared HPC films with an average thickness of 208 ± 11 μm by solvent-casting.

### 3.2 Thermal Stability

Differential scanning calorimetry (DSC) was performed on HPC films and fibers to determine any thermal transitions. The DSC traces, which are presented in Figure 3a, show broad endothermic peaks with maxima at 80 °C (fibers) and 98 °C (films), which we assign to the loss of water. The DSC traces also reveal very weak endothermic peaks at 200 ± 0.6 °C (fibers) and 197 ± 0.2 °C (films), which may be associated with the melting process of HPC, as reported by Rials and Glasser.^13^ The authors observed a small endothermic signal over a broad range of temperatures, from 165 to 210 °C, correlated with HPC melting. No glass transitions can be discerned, possibly because of overlap with the water-loss signals.

**Figure 3:**
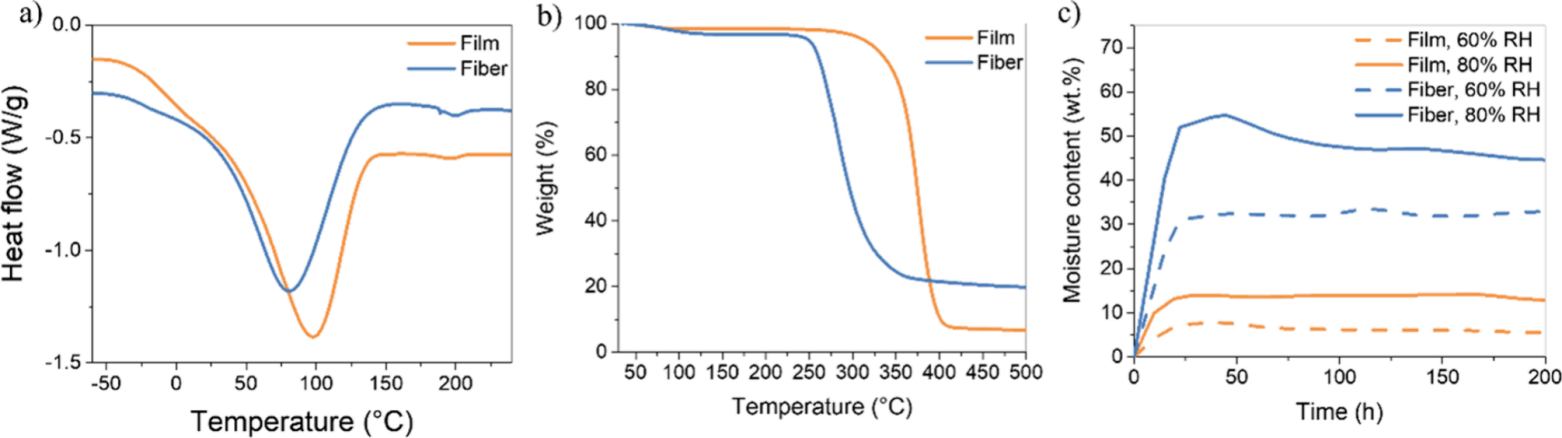
Representative (a) DSC traces (exo up) and (b) TGA traces of an HPC film (orange) and a fiber (blue). Measurements were performed under nitrogen with a heating rate of 10 °C/min. (c) Representative moisture content of HPC fibers (blue) and films (orange) at 60% RH (dotted lines) and 80% RH (solid lines) as a function of time.

Thermogravimetric analysis (TGA) was used to investigate the thermal stability of the HPC fibers and films (Figure 3b). We attribute the initial weight loss observed upon heating to ca. 150 °C to the loss of moisture. The water content of films (after drying and storage under dry conditions) varied from 0.2 to 2.8 wt %, while that of HPC fibers ranged from 2 to 3.8 wt %. These values indicate that even after extensive drying, modest water uptake cannot be avoided, possibly due to adsorption of environmental moisture during handling. This is consistent with the fact that the high-surface-area fibers display a higher moisture content than the films.

A comparison of the TGA traces shows that the fibers display a lower thermal degradation temperature and a higher char yield than films (Figure 3b). T10%, i.e., the temperature at which the mass loss is 10%, is 260 ± 1 °C for HPC fibers and 335 ± 6 °C for HPC film; the latter value matches previous reports.^14,39^ The char yields at 500 °C are 20 wt % for fibers and 7 wt % for films, which suggests the presence of up to ca. 13 wt % of residual CaCl_2_ in the fibers. The reduced thermal stability of the HPC and increase in char yield appear to be a direct consequence of these residues and match previous findings on CaCl_2_-impregnated cellulose fibers and cellulose/PVA/alginate fiber prepared by wet spinning in a CaCl_2_ bath.^40,41^ The presence of CaCl_2_ in the fibers was confirmed by EDX analysis (Figure S1 and Table S2, Supporting Information). The investigation shows the presence of calcium and chloride atoms in the fibers and reveals a CaCl_2_ content of ca. 2.5 wt %. We note that the high water solubility of neat HPC prevents reducing the CaCl_2_ content in the fibers further, but the thermal stability still exceeds 260 °C and is by far sufficient for the applications considered here. As we will discuss later, the presence of the CaCl_2_ seems to contribute considerably to the improved mechanical properties of the fibers.

### 3.3 Water Sorption of HPC Films and Fibers

Moisture uptake measurements were conducted for HPC fibers and films at 23 °C and 60 as well as 80% RH (Figure 3c). After 3 days, all samples reached their equilibrium moisture content. Average moisture uptake values were determined from three replicates, and weighing was extended for 5 days after the equilibrium was reached.

A small overshoot in the moisture content of fibers is observed during the first 48 h at 80% RH, followed by a constant decrease in moisture content until stabilization. This phenomenon, previously mentioned in the literature,^42,43^ is observed in highly hygroscopic materials under high-humidity conditions. At high relative humidity, the high surface area of the fibers encourages rapid water sorption at the surface, forming water clusters at the surface.^44^ Once the surface reaches saturation, the excess water is released from loosely bound outer water layers, resulting in a slight decrease in moisture content over time. This behavior is consistent with the water sorption behavior of cellulosic materials.

A significant difference in moisture content was observed between fibers and films due to the higher surface area of fibers and the residual CaCl_2_ in the former. At 60% RH, the equilibrium moisture content of HPC fibers reached 30 ± 2 wt %, while that of HPC films was 6 ± 2 wt %, which is consistent with a reported value of ca. 4 wt % at 50% RH for HPC films.^45,46^ At 80% RH, a similar behavior was observed; the equilibrium moisture content of HPC fibers reached 39 ± 10 wt %, while a value of 13 ± 1 wt % was determined for films. Again, the result for HPC films is aligned with a reported equilibrium moisture content of ca. 20 wt % at 90% RH.^45,46^ At both relative humidities, the fibers exhibit a higher moisture content than the films. This difference can be explained by the residual CaCl_2_ in the fibers, as mentioned above. Despite representing ca. 2.5 wt % of the fibers, the high hygroscopicity of CaCl_2_ could largely contribute to the fiber mass uptake, as reported by Sakara and Yamaguchi.^47^ The authors reported an increase in moisture content from 4 to 15 wt % upon incorporating 3 wt % CaCl_2_ in hydroxypropyl methylcellulose. Moreover, the residual CaCl_2_ content may also be responsible for the overshoot in water uptake observed at 80% RH for fibers. At such high RH, CaCl_2_ absorbs enough water to dissolve, leading to a loss of material in the fiber and a decrease in moisture content after 48 h.

### 3.4 Degree of Crystallinity

The degree of crystallinity (D.o.C.) of HPC fibers and films was evaluated based on wide-angle X-ray scattering (WAXS) data. Representative WAXS patterns are shown in Figure 4. The WAXS patterns reveal two peaks at 2θ ca. 8° and 2θ ca. 20° for both HPC fibers and films. The analysis of the data afforded D.o.C. values of 44 ± 1% for fibers and 43 ± 1% for films. We report the D.o.C. rather than the crystallinity index (CI), as D.o.C. provides a more quantitative and physically meaningful measure of the crystalline fraction within the material.^48^ Cremer et al. reported similar peaks on X-ray diffraction patterns with a CI of ca. 71%.^49^ Such high crystallinity in HPC films was correlated with the significant differences between HPC with identical molecular weight but presenting different distributions of substituents along the chains. These structural differences have a direct impact on the physicochemical properties of HPC materials, as reported by the authors and could increase the crystallinity of HPC.

**Figure 4:**
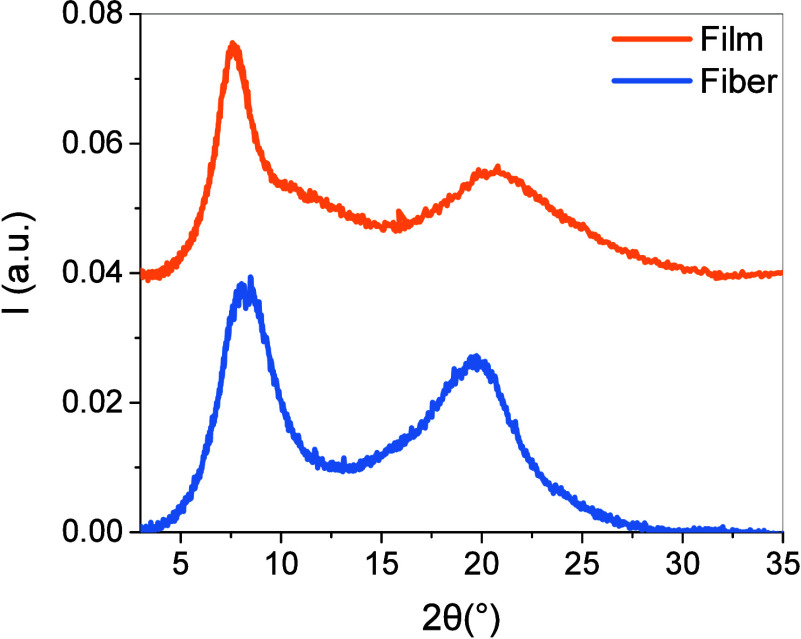
Wide-angle X-ray scattering (WAXS) patterns of HPC film (orange) and fiber (blue). The patterns are shifted vertically for clarity.

Overall, using the same HPC reference, the data revealed that the morphology and crystallinity of the wet-spun fibers are comparable to those of solvent-cast films.

### 3.5 Alignment of HPC Chains

Polarized optical microscopy (POM) images were recorded to assess the arrangement of HPC chains in solvent-cast films and wet-spun fibers. The samples were placed between two crossed linear polarizers, and the corresponding images of HPC films and fibers under normal light and cross-polarized light are presented in Figure 5. The HPC film shows a very slight birefringence under cross-polarized light, while the HPC fiber presents bright colors indicative of anisotropy. This observation highlights HPC chain alignment under the shear forces inherent to wet spinning, which could also contribute to better mechanical resistance.

**Figure 5:**
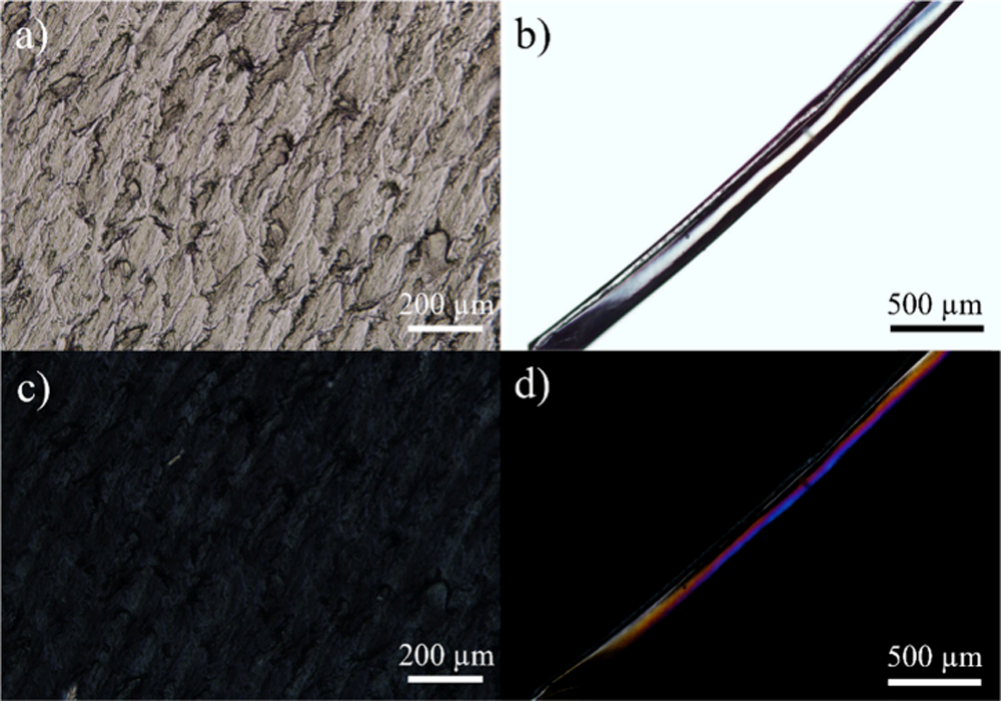
POM images of HPC films (a, c) and HPC fibers (b, d) under normal light (a, b) and under cross-polarized light (c, d).

### 3.6 Mechanical Properties

Dynamic mechanical analysis (DMA) of HPC fibers and films stored at 0% RH was carried out to characterize their thermomechanical behavior. Figure 6a shows representative traces of the storage modulus *E*′ and the loss modulus (tan δ) traces as a function of temperature. Key parameters extracted from the DMA experiments are presented in Table 2.

**Figure 6:**
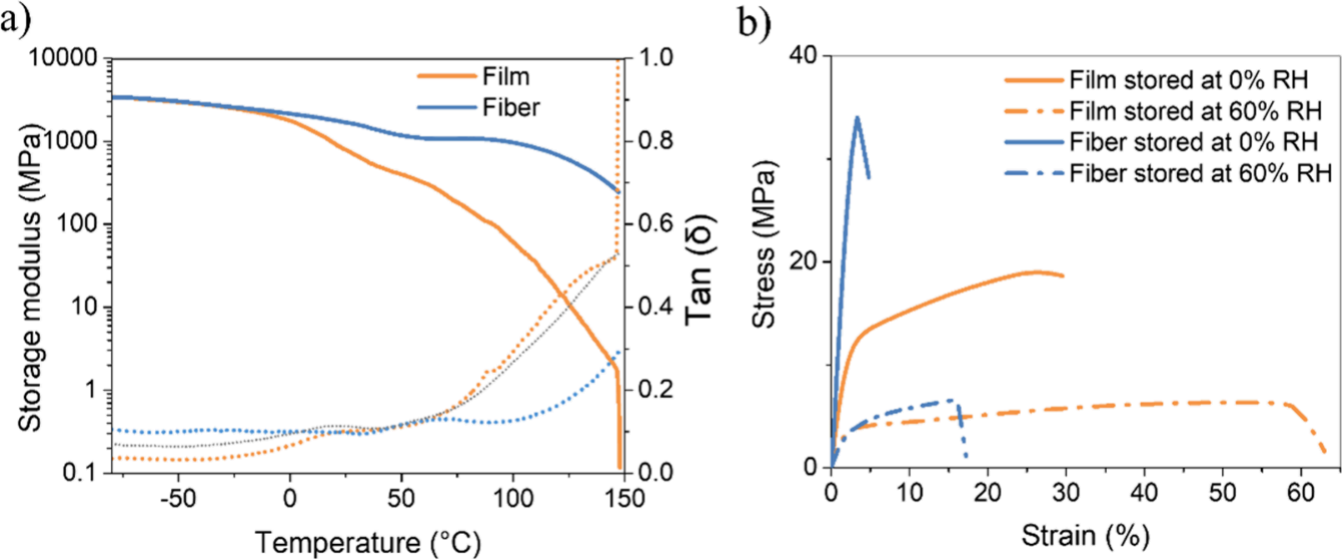
(a) Representative DMA traces showing the storage modulus (solid lines) and the loss tangent (dotted lines) of HPC fibers (blue) and films (orange) stored at 0% RH. The measurements were performed at a frequency of 1 Hz and a heating rate of 3 °C/min. (b) Representative stress–strain curves of HPC fibers (blue) and films (orange) stored at 0% RH (solid lines) and after being conditioned for at least a week at 60% RH (dotted lines). The measurements were conducted at room temperature with a strain rate of 10 mm/min.

**Table 2:** Thermal and Mechanical Properties of HPC Fibers and Films

	HPC fibers		HPC films					
storage modulus at −80 °C (GPa)^a^	3.4 ± 1.5		3.4 ± 1.9					
storage modulus at 25 °C (GPa)^a^	1.7 ± 0.7		0.7 ± 0.5					
*T*_g_ (°C)^a^	66 ± 3		n.d.^c^					
storage conditions	0% RH	60% RH	0% RH	60% RH	0% RH CaCl_2_			
					2.5 wt %	5 wt %	10 wt %	15 wt %
Young’s modulus (MPa)^b^	1346 ± 148	258 ± 76	657 ± 44	272 ± 20	972 ± 180	1203 ± 228	790 ± 165	1125 ± 135
tensile strength (MPa)^b^	36 ± 6	8 ± 1	19 ± 1	4.2 ± 0.6	14 ± 2	8 ± 3	14 ± 3	5 ± 1
strain at break (%)^b^	4 ± 1	19 ± 5	27 ± 2	62 ± 14	5 ± 1	2 ± 1	2 ± 1	2 ± 1

a Determined by DMA. Samples were stored at 0% RH.

b Determined by tensile tests at room temperature with a strain rate of 10 mm/min. Samples were conditioned as indicated.

c Not detectable.

At −80 °C, where HPC is in its glassy state, fibers and films exhibit the same *E*′ of 3.4 GPa. The storage modulus of HPC films decreases continuously with temperature and assumes a value of 0.7 ± 0.5 GPa at 25 °C. The corresponding loss tangent curve (tan δ) of HPC films (Figure 6a) reveals a first relaxation between 0 and ca. 50 °C and a second relaxation that sets in at ca. 50 °C and extends until failure at ca. 150 °C. The DMA traces suggest that the first relaxation is related to a glass transition, which is in agreement with previous observations reported in the literature,^13,14^ but due to the overlap of the two relaxation processes, it is difficult to pinpoint the exact glass transition temperature (*T*_g_). The gradual decrease of *E*′ above *T*_g_ is steeper and likely associated with the melting of crystalline domains and the breaking of intermolecular hydrogen bonds over a broad temperature range, as observed by Charlet and Gray for HPC films.^50^

Below ca. 0 °C, the DMA traces of the fibers mirror those of the films, but above this temperature, *E*′ drops much less. Thus, at 25 °C, a significantly higher storage modulus of 1.7 ± 0.7 GPa than in the films (0.7 ± 0.7 GPa) is observed, while at 150 °C, where the films fail, *E*′ has only decreased to 231 ± 20 MPa. The corresponding loss tangent curves (tan δ) of the HPC fibers show a first relaxation with a maximum at 66 °C, which we attribute to *T*_g_. A second transition sets in at ca. 100 °C. This onset is considerably higher than the one observed for the films, and the magnitude of this relaxation is also less pronounced. We speculated that this considerable difference is in part related to a higher degree of alignment in fibers, as observed by microscopy (Figure 5). The wet spinning processing conditions improved the chain alignment and thus caused an increase in strength and stiffness in the direction of the fiber axis. Moreover, the presence of residual CaCl_2_ in the fibers could also play a role, as it was previously reported that CaCl_2_ increases the strength and stiffness of regenerated cellulose, on account of physical cross-links that arise from the coordination of the Ca^2+^ ions to the cellulose (vide infra).^51^ To test if this effect is also at play in the present HPC fibers, we solvent-cast HPC films containing ca. 1.6 wt % CaCl_2_ (see Section 2 for details). The corresponding DMA traces, presented in Figure S5 (Supporting Information), show the same relaxation processes as observed for the neat HPC films. Nevertheless, in the glassy state, *E*′ is considerably higher (7.1 ± 0.1 vs 3.4 ± 1.9 GPa at −80 °C) and the drop in *E*′ across the first transition (0–ca. 50 °C) is less pronounced than in the neat HPC films. Thus, at 25 °C, *E*′ of the CaCl_2_-containing HPC films (1.9 ± 0.2 GPa) is substantially higher than that of the CaCl_2_-free samples. However, in the temperature range associated with the second relaxation process (50–ca. 150 °C), the modulus drop of the CaCl_2_-containing samples mirrors that seen for the CaCl_2_-free films, reflecting that the stiffening effect of CaCl_2_ is lost at higher temperatures. Thus, at least at elevated temperatures, the high stiffness displayed by the fibers is not primarily related to the presence of the salt, but instead driven by the uniaxial orientation of HPC.

We also conducted tensile tests to investigate the mechanical performance of HPC fibers and films further, particularly concerning the effect of CaCl_2_. Figure 6b shows representative stress–strain curves of HPC fibers and CaCl_2_-free films, and the mechanical data are compiled in Table 2. Tensile tests were conducted at 23 °C on samples stored at 0% RH and 60% RH to explore how moisture affects the mechanical properties. HPC films stored in a dry environment exhibit a Young’s modulus of 657 MPa; they yield at a strain of ca. 4% and show plastic deformation with failure occurring at a strain of ca. 27% and stress at break of 19.1 MPa. Their behavior matches the one reported previously in the literature.^14^ The dry HPC fibers are much stiffer (Young’s modulus = 1.35 GPa) and stronger (tensile strength = 35.7 MPa) than the films, and instead of yielding, they show brittle fracture with a strain at break of ca. 3.5%. The stress–strain curves of the CaCl_2_-containing films (Table 2 and Figure S3 in the Supporting Information) show that their Young’s modulus increased up to 1.2 GPa for a CaCl_2_ content of 5 wt %, while their tensile strength decreases to 5–14 MPa, and the elongation at break decreases to 2–5%. In comparison to CaCl_2_-free films, the stiffness of CaCl_2_-containing films is improved, while their strength is reduced, which reflects that the high strength displayed by the fibers is not primarily related to the presence of CaCl_2_. Indeed, previous studies on wet-spun fibers of different cellulosic materials reported an increase in strength due to an improved chain alignment that the shear forces applied during wet spinning induce.^52–54^ Iwamoto et al. compared wet-spun fibers and solvent-cast films made of wood and tunicate nanofibers (CNF) and reported a random orientation of CNF in the horizontal plane of the film but a clear orientation in the fibers, demonstrating the impact of wet spinning shear forces on the alignment of polymer chains.^53^ The CNF fibers exhibited a Young’s modulus of 5.5–23.6 GPa, a tensile strength of 90–406 MPa, and a strain at break of 1.5–5.6%. The much higher stiffness and strength of these fibers is ascribed to the high extent of uniaxial orientation of the cellulose molecules within the CNFs, along with a better alignment of these nanofibers in the wet spinning process. Similar results were reported by Lundhal et al.,^32^ who spun TEMPO-oxidized CNF fibers from water and reported a Young’s modulus of 8.6–21.3 GPa, a tensile strength of 136–326 MPa, and a strain at break of 2.8–8.4%, depending on the extent of orientation. Our work demonstrates similar effects (Figure 5) with a random orientation of HPC chains in solvent-cast films but an anisotropic arrangement of the polymer chains in wet-spun fibers, as confirmed by POM.

Tensile tests carried out after conditioning the samples at 60% RH reveal qualitatively similar outcomes for films and fibers that are indicative of water-induced plasticization (Figure 6b and Table 2). In both cases, the tensile tests show a reduction in the Young’s modulus and tensile strength vis-à-vis the dry samples, while the elongation at break is increased. The mechanical contrast between dry and humid states is a bit more pronounced for the fibers. Nevertheless, the fibers remain slightly stiffer and stronger than the films, which may be related to chain orientation. Importantly, the data show that while the mechanical properties of HPC films and fibers are strongly affected by the environmental conditions, both films and fibers retain mechanical properties that are suitable for use in cosmetic products, wound dressing, or flushable products at a RH of up to 60%, while they are fully soluble when immersed in water (Figure S4, Supporting Information).

## 4 Conclusions

For the first time, hydroxypropyl cellulose (HPC) fibers were successfully produced using a rapid, sustainable, and scalable wet spinning process. By a systematic optimization process, including dope concentration, type of coagulation bath, flow rate, and temperature, we identified the most effective approach to obtain defect-free fibers with high stiffness. Moreover, this technology requires only water as a solvent and a salt coagulation bath, offering an environmentally friendly approach to producing transparent and uniform HPC fibers. While the thermal stability of the fibers is lower than that of solvent-cast HPC films, their degradation temperature (ca. 260 °C) remains comparable to that of many conventional polymers. HPC fibers also demonstrated higher water sensitivity than films. Both thermal stability and water sensitivity are affected by the presence of residual salt from the coagulation step, which also affects the mechanical properties. While the presence of residual CaCl_2_ can enhance the stiffness of HPC fibers, the superior mechanical performance observed for the fibers, even under high RH, is mostly assigned to the shear forces inherent to wet spinning. HPC fibers presented intense birefringence under cross-polarized light, indicating that wet spinning promotes chain alignment and enhances chain interactions in the fibers. Overall, the HPC fibers exhibit improved mechanical properties, good thermal stability, and a degree of crystallinity that is similar to the HPC films. Under both dry and humid conditions, the performance of HPC fibers is better than that of the films, positioning them as promising candidates for applications in biomedical, sanitary, and flushable products.

## Supplementary Material



## Notes

Additional experimental details for HPC spinning optimization, and characterizations, including EDX analysis of HPC films and fibers, ^1^H NMR spectrum of HPC, stress–strain curve and DMA traces of HPC films containing CaCl_2_, and time-lapse pictures of HPC films and fibers immersed in water (PDF)
