# Supplementary material for: Wet Spinning of Sustainable Hydroxypropyl Cellulose Fibers
Source: Biomacromolecules. 2025 Nov 25;26(12):8828–36. doi: 10.1021/acs.biomac.5c02062 (PMC12690520; doi:10.1021/acs.biomac.5c02062)
Supplement: Supplementary file 1 [file bm5c02062_si_001.pdf]

Wet spinning of sustainable hydroxypropyl cellulose fibers

Manon Guivier[a,b], Christoph Weder[a,b*]*

[a]Adolphe Merkle Institute, Polymer Chemistry and Materials, University of Fribourg, 1700 Fribourg, Switzerland

[b]NCCR Bio-inspired Materials, University of Fribourg, 1700 Fribourg, Switzerland

manon.guivier@unifr.ch

christoph.weder@unifr.ch

Supplementary Information

Table S1. Summary of wet spinning processing parameters investigated, and the corresponding results (optimized results are in bold). A flow rate of 0.5 mL/min, a rotating speed of 25 rpm, and a 19G needle were used and fixed during the optimization.

	**Bath**	**Water**	**$CaCl_2$**	**NaCl**	**HCl**	**2-butanol**	**2-propanol**	**Acetone**	**DMAc:water (1:1)**
**Temperature (°C)**		23-80	23-80	23-80	23	23	23	23	23-50
**HPC concentration (wt.%)**	5	N.F.	N.F.	N.F.	N.F.	N.F.	N.F.	N.F.	N.F.
	8	N.F.	U	U	S/U	S/U	S/U	S/U	U
	10	**F**	**F**	U	S	S	S	S/U	U
	12	**F**	**F**	U	S	S	S	S/U	U
	15	Too viscous							
	20	Too viscous							
**HPC 12 wt.%**									
**Temperature (°C)**	23	N.F.	U	U	S	S	S	S/U	U
	50	N.F.	U	U	-	-	-	-	U
	70	U	**F**	**F**	-	-	-	-	-
	80	U	**F**	**F**	-	-	-	-	-
**HPC 12 wt.%, 70°C**									
**Coagulation bath concentration (wt.%)**	2	-	U	U	-	-	-	-	-
	4	-	**F**	**F**	-	-	-	-	-
	8	-	**F**	**F**	-	-	-	-	-

N.F.: no fibers, S: shrinkage, U: unstable outside of bath, F: homogeneous fibers

Table S2. EDX analysis of HPC fibers and $CaCl_2$-free film.

		HPC fibers	HPC films
Weight (%)	C	66.8 ± 0.2	59.5 ± 0.2
	O	30.8 ± 0.2	40.5 ± 0.2
	Cl	1.4 ± 0.1	-
	Ca	1 ± 0.1	-
Atomic (%)	C	73.6 ± 0.1	66.2 ± 0.2
	O	25.5 ± 0.1	33.8 ± 0.2
	Cl	0.6 ± 0.1	-
	Ca	0.3 ± 0.1	-

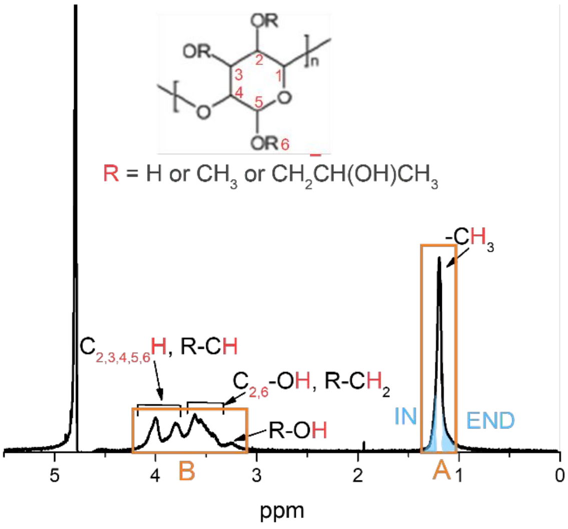

Figure S1. ^{1}H NMR spectrum (CDCl3, 400 MHz) of HPC.

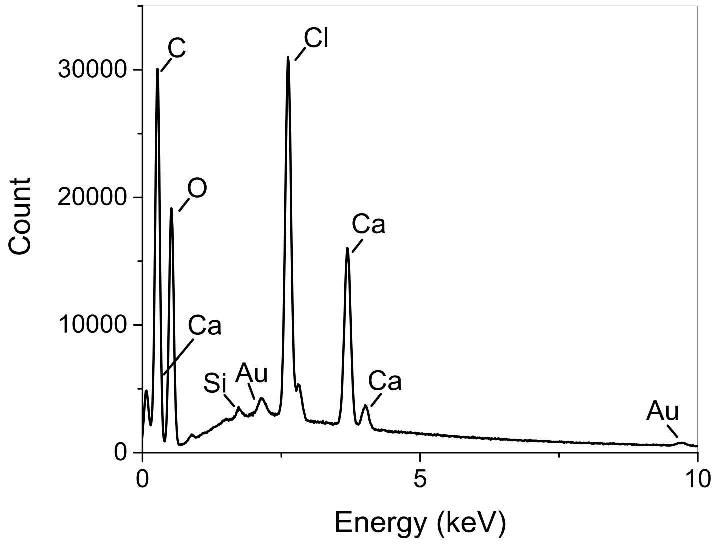

Figure S2. EDX spectrum of HPC fibers.

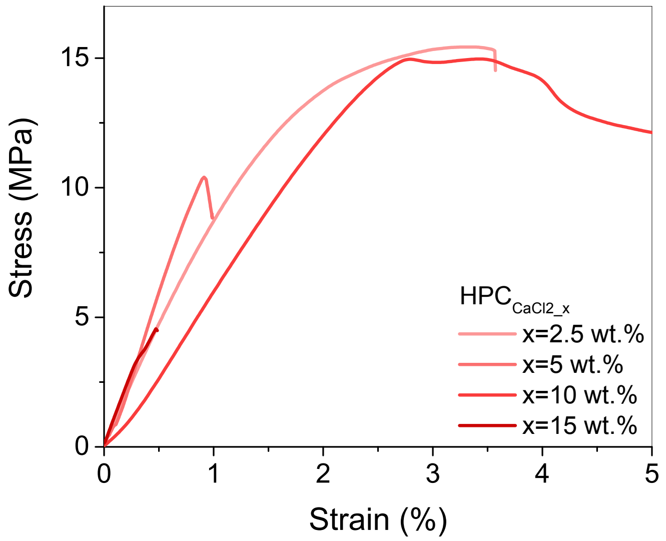

Figure S3. Representative stress-strain curves of HPC films containing 2.5 wt.%, 5 wt.%, 10 wt.% and 15 wt.% of $CaCl_2$ stored at 0% RH. The measurements were conducted at room temperature with a strain rate of 10 mm/min.

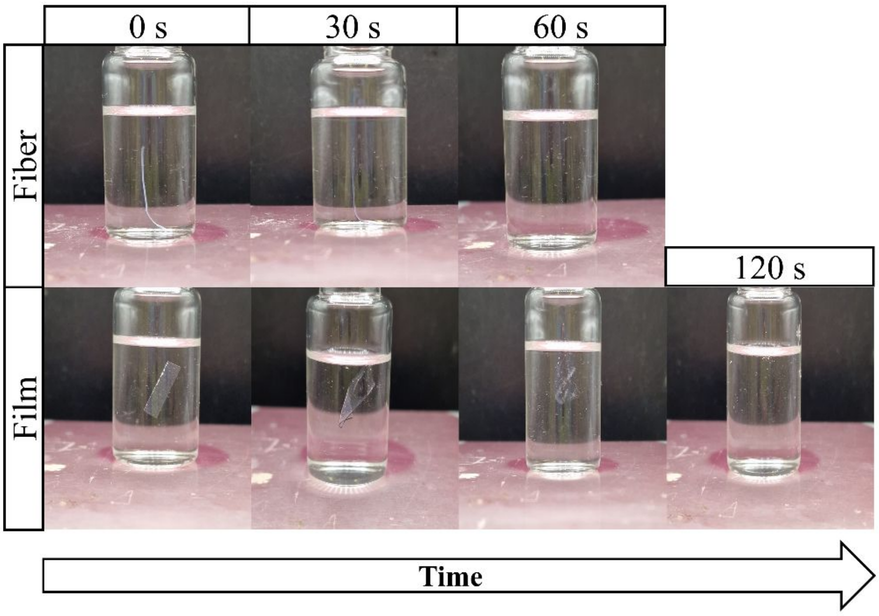

Figure S4. Time-lapse pictures of HPC fibers and films immersed in water.

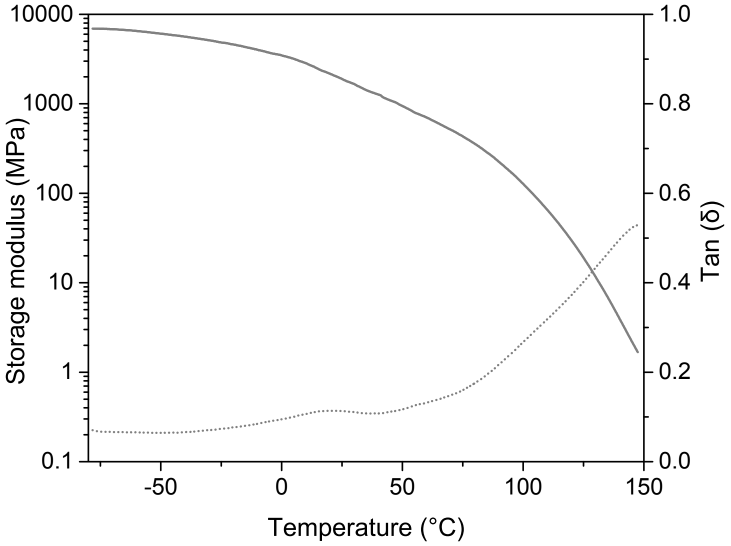

Figure S5. Representative DMA traces showing the storage modulus (solid line) and the loss tangent (dotted line) of HPC films containing 1.6 wt.% $CaCl_2$ stored at 0% RH.
